# Visualizing Ruby Emission Decay Lifetime with Slow-Motion Digital Cameras: A Demonstration for Students

**DOI:** 10.1021/acs.jchemed.4c01529

**Published:** 2025-04-07

**Authors:** Dinesh Dhankhar

**Affiliations:** Los Alamos National Laboratory, Los Alamos, New Mexico 87545, United States

**Keywords:** First Year Undergraduate/General, Analytical Chemistry, Hands on Learning/Manipulations, Photochemistry, Kinetics, Fluorescence Spectroscopy, Molecular Mechanics/Dynamics, Instrumental Methods

## Abstract

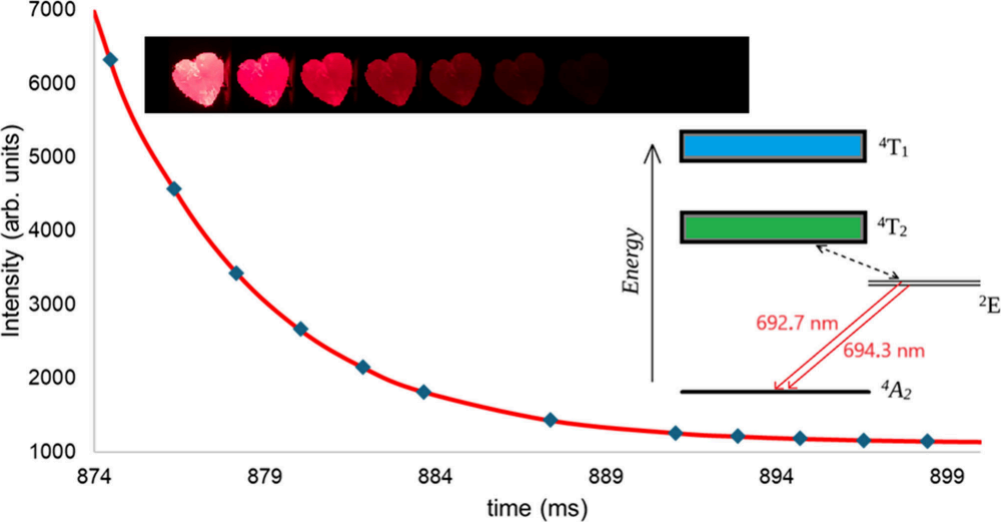

This short article describes a safe way of observing the emission decay from a ruby crystal using a photographic flash. This demonstration does not require the use of lasers and can be easily and safely performed in a classroom or a home setting. The ruby sample was optically excited with a short-duration photographic flash, and the subsequent relaxation of the excited state population through intermediate metastable states was monitored using three different types of slow-motion digital cameras. Monitoring the recorded video frames allowed visualization of the population decay kinetics. In addition, the measurement of frame intensities allowed for the computation of the excited state lifetime and decay rate constant. This demonstration can be utilized in several different chemical education courses at the advanced high school or undergraduate level, for example, in the courses that involve concepts related to photochemical reaction kinetics, flash photolysis, lasers, etc.

The concept of the lifetime of excited states is important for several scientific disciplines, such as lasers, photochemistry, quantum physics, and chemistry etc. These concepts also apply to popular fields, such as fluorescence lifetime imaging and microscopy. Here, an easy demonstration is presented that allows students to observe visually the emission decay lifetime in ruby crystals with easily available off-the-shelf items.

Ruby (aluminum oxide doped with chromium ions) is an important system. The world’s first laser used ruby as its gain medium.^1^ Several student experiments have been reported in the literature for measuring the decay time and the spectrum of ruby emission,^2−4^ including in a major physical chemistry textbook.^5^ In addition, time-resolved spectroscopy experiments for chemical education have been previously reported.^6−9^ While some of the previous activities and experiments use commercial spectrofluorimeters capable of lifetime measurements, many others use the excitation sources that range from pulsed lasers (Nitrogen (N_2_) or Neodymium YAG (Nd:YAG)), pulsed LEDs, electronically switched or physically chopped continuous wave laser or arc light sources etc. Some of them also make use of fast electronic detectors, such as photomultiplier tubes, requiring high voltage power supplies, while others make use of single photon counters, silicon photomultipliers, or home-built transimpedance amplifier photodetector circuits.

The demonstration presented here, on the other hand, only makes use of photographic flash used in day-to-day photography, and a slow-motion-capable digital camera to visualize the ruby emission decay. The slow-motion-capable camera could simply be a cellphone camera capable of recording a slow-motion video, such as 240 frames per second, or a stand-alone digital camera, such as amateur astronomy hobby cameras, which are available at low cost. Imaging devices and cameras have been an important tool for scientific education, and several previous articles have made use of these tools.^10−16^

## Brief Theoretical Description of Origin and Decay of Ruby Emission

**Figure 1:**
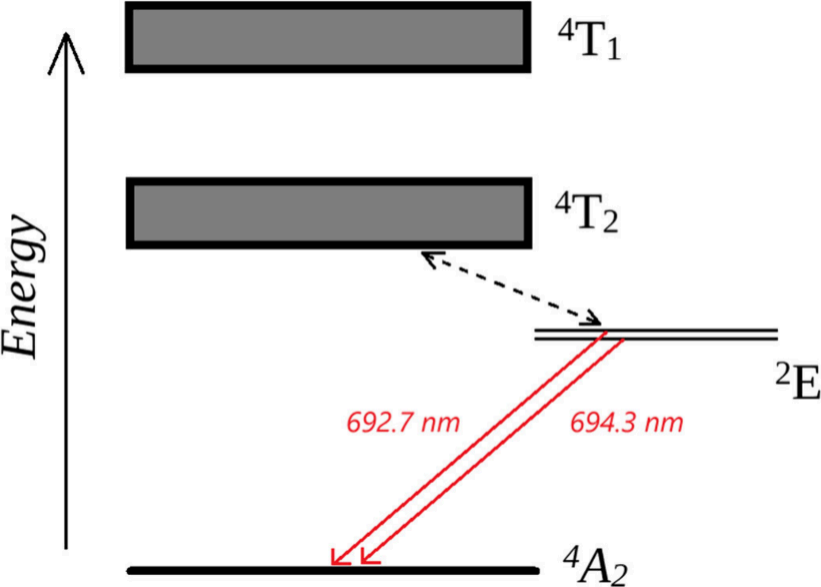
Energy level diagram of ruby and some of the electronic transitions therein.

Ruby consists of chromium (Cr^3+^) ions doped into an aluminum oxide lattice (Al_2_O_3_). Chromium, which is a transition metal, has partly filled d orbitals with equal energies. When doped Cr^3+^ ions replace some of the Al^3+^ ions in the lattice, the interaction with the lattice causes the degenerate d levels to split in their energy, forming certain bands.^3,5,17−19^ The resultant energy level diagram is shown in Figure 1. The broad absorption bands of ruby in the green and blue-violet regions of the spectrum are due to transitions from the ground state (^4^A_2_) to the ^4^T_2_ and ^4^T_1_ bands. These absorption bands are responsible for the efficient pumping of ruby with broad-spectrum xenon flash lamps. After excitation into the ^4^T_2_ and ^4^T_1_ bands, the excited species nonradiatively relax into a metastable state, ^2^E.^3,5,17−19^ This relaxation happens very quickly, within nanoseconds. The transition from the ^2^E state to the ground state is radiative with a lifetime of a few milliseconds. The ^2^E energy level is split into two, giving rise to two emission lines in the spectrum at approximately 692.7 and 694.3 nm wavelengths. The stronger of these two, at 694.3 nm is also the emission line of the ruby laser. It is important to note that the exact wavelengths and the split between these lines change as a function of pressure and temperature, enabling the use of ruby for high-pressure and temperature sensing.^20−22^

### Kinetic Law of Fluorescence Decay

When an excited state decays by emitting light (fluorescence, phosphorescence, etc.), the intensity of the emission is directly proportional to the population of species (atoms/molecules) in the excited state (N). The population of the species in the excited state can be computed by knowing that the population rate of decay is directly proportional to the population at a given time (eq 1). The proportionality constant in eq 1, *k*, is known as the decay rate constant, and the solution of this equation represents an exponential decay of population as a function of time (eq 2) from the excited state. Here, N(t) is the excited state population at time t, and N(0) is the initial excited state population at time t = 0.
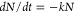
1
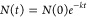
2

As the population in the excited state decreases, so does the intensity of the emission. Intensity decay of emission can thereby be mathematically described by eq 3 below. Here, I(t) is the intensity of emission at time t, and I(0) is the intensity of emission at time t = 0.
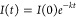
3The constant *k* has the unit of s^–1^ and can also be represented as inverse of a time constant
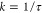
4which gives rise to the more familiar form of eq 3, with *k* written in the form of a time constant (eq 5).
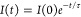
5τ, the time constant, is also known as the lifetime of the excited state. In a time equal to τ, the intensity of emission decreases to approximately 37% of its initial value. Many simple fluorophores follow such a single exponential decay. However, complex molecules, or a molecule in a more complex environment may have emission decay that is described by a sum of several single exponential decays.

## Experimental Setup Details

### Sample

The ruby sample used in these experiments was a ruby earring made of synthetic ruby. Any other ruby sample, such as ruby balls used in industry or a ruby rod from a used ruby laser, can also be used. Synthetic rubies are a good choice for this demonstration as they cost significantly less than their natural counterparts. In addition, synthetic rubies are generally very pure. Most rubies utilized for scientific and industrial purposes are also synthetic.

### Photoexcitation of the Sample

To excite the emission in the ruby sample, a photographic flash (Sunpak auto 422 D Thyristor) was used at the lowest power setting (1/16th of the maximum power). According to the manual of the flash,^23^ at that setting, the flash duration is 1/12500 of a second (∼80 μs), much smaller than typical ruby emission decay time constant of ∼ 3.5 milli-seconds. Other flashlights, such as built-in flashes in a Single Lens Reflex (SLR) or Digital Single Lens Reflex (DSLR) camera, can also be used. Typically, the flash duration of flash on SLR/DSLR cameras is several times shorter than 1 ms when used at their lowest power setting, which would suffice for visualizing the decay of ruby emission.

In addition, the xenon flashes used in cameras produce a significant amount of blue and near-UV light, which is efficiently absorbed by the ruby samples. For reference, the emission and absorption spectrum of ruby is shown here.^17^

If required, excitation and emission filters in front of the photographic flash and the recording camera, respectively, may be utilized to eliminate wavelength interference. These filters could be simple gel color filters, for example. A blue or green get filter may be utilized as an excitation filter, and a red gel filter may be used as an emission filter.

### Recording of Emission Decay from the Ruby Sample

The emission from the ruby sample was captured with a slow-motion digital camera after flash excitation. The three different digital cameras used in the experiments were as follows: first, a Casio Exilim FH20 camera, which can record at 1000 frames per second, was used. The Casio Exilim high-speed camera was chosen because this brand of camera is available at a relatively low cost on the used market, and 1000 frames per second allows a good recording of the decay of ruby’s emission. Second, a cellphone camera (Google Pixel 4), which can record slow-motion videos at a frame rate of 240 frames per second, was utilized. Experiments with the cellphone camera were performed due to its ubiquitous availability in the student community. Third, an amateur astronomy camera, a ZWO ASI662MC, was used. This camera can record at different frame rates depending on the resolution of the image. More importantly, it can record raw video frames without any compression. For experiments with this camera, raw frames were recorded at approximately 530 frames per second at a resolution of 100 pixels x 200 pixels. SharpCap software (free to download and use) was used to interface with and record data from the ZWO ASI662MC camera.

**Figure 2:**
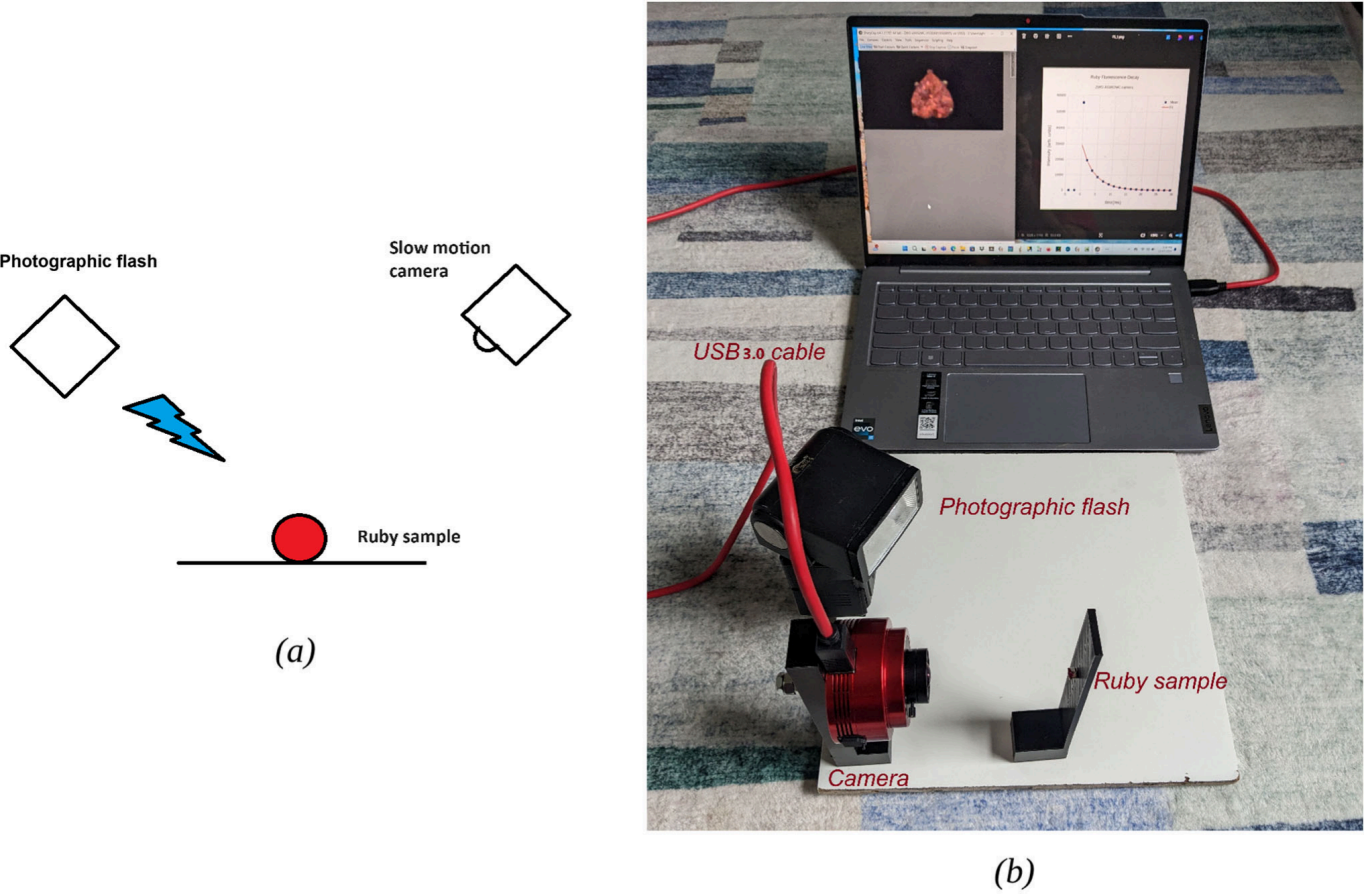
(a) Simplified block diagram of the experimental setup and (b) a picture of the actual setup with a ZWO ASI662MC camera.

A block diagram of the experimental setup is shown in Figure 2(a). The ruby sample was illuminated from the side using a photographic flash, as shown in Figure 2(b). The camera shown in Figure 2(b) is the ZWO ASI662MC, which transmits the data via a USB 3.0 cable to the laptop. Raw video frames were recorded on the laptop at a rate of approximately 530 frames per second. For the video recording with a cellphone camera and a Casio Exilim camera, the data were recorded in the device memory and later transferred to the laptop for analysis.

The setup shown in Figure 2(b) can also be constructed using 3D printed parts. 3D design files and assembly instructions can be found in the Supporting Information (STL files for 3D printing the experimental setup). In addition, two videos showing the assembly, experiment, and data analysis steps are also available in the Supporting Information (Experiment Video 1 and Experiment Video 2).

### Data Analysis Steps

Detailed step-by-step instructions for data analysis are provided in the Supporting Information and in the demonstration videos. Briefly, the recorded slow-motion video frames from the cellphone and Casio Exilim videos were extracted using Planetary Image Pre Processor (PIPP) software (free to download and use). For data recorded using the ASI662MC camera, the raw.fits frames were binned 2×2 to debayer the frame (i.e., to eliminate the effect of color filter matrix on the sensor) and then analyzed using ImageJ software. To measure the decay time constant of the emission, the extracted frames intensities in the region of interests were measured using ImageJ software and plotted as a function of time. The accurate frame time was extracted from the.fits frames’ metadata information.

## Results and Discussion

### Visualizing the Emission Decay

**Figure 3:**
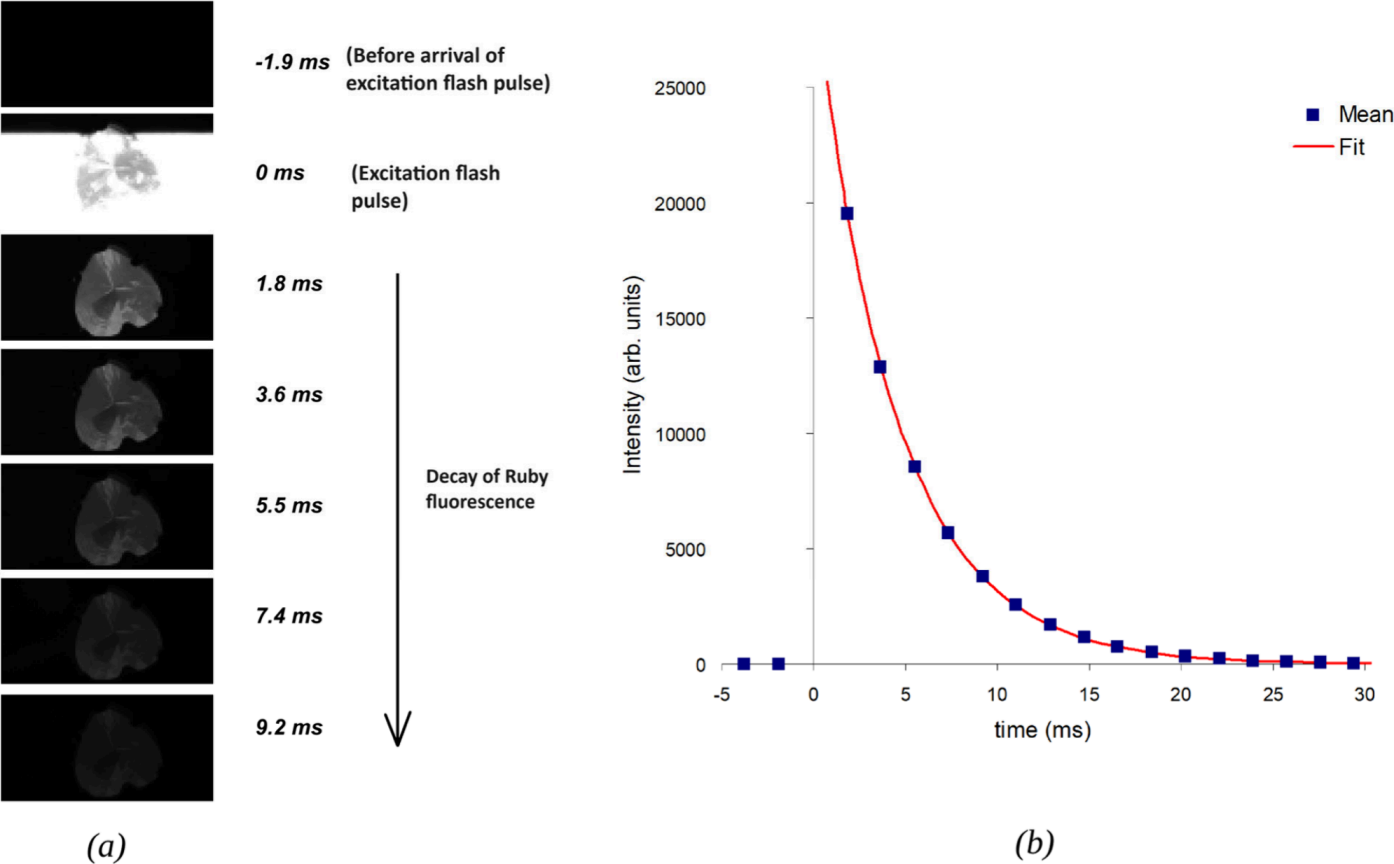
(a) Emission decay of ruby as captured in slow-motion video frames from a ZWO ASI662MC camera at ∼ 530 frames per second. (b) Plot of emission decay profile after exciting the ruby sample with a photographic flash. The plotted emission intensity data were measured from the slow-motion video frames of ZWO ASI662MC camera. Time constant computed was ∼ 4.5 ms by fitting a single exponential decay profile to the measured data.

**Figure 4:**
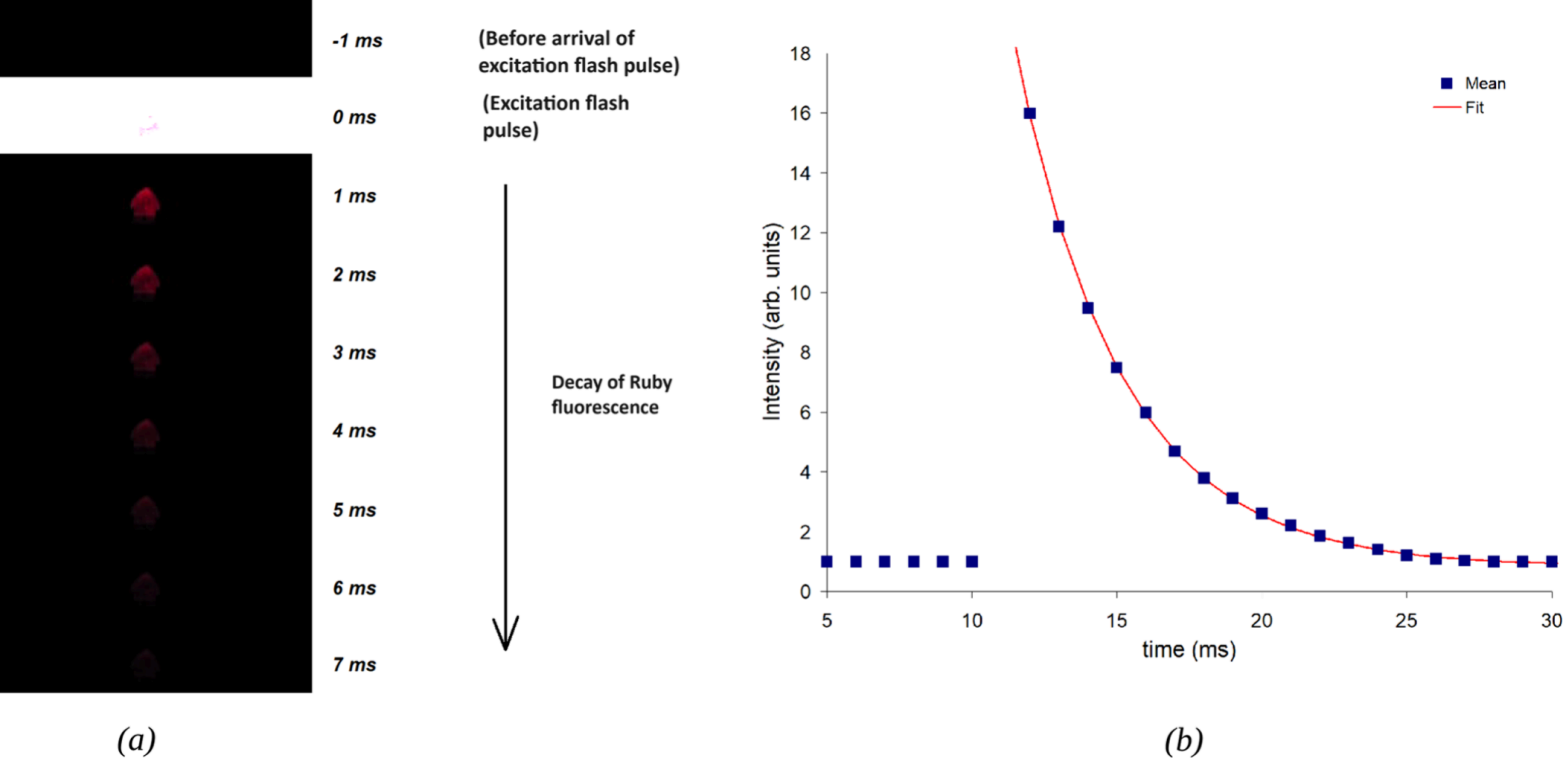
(a) Emission decay of ruby as captured in slow-motion video frames from a Casio Exilim FH20 camera at 1000 frames per second. (b) Plot of emission decay profile after exciting the ruby sample with a photographic flash. The plotted emission intensity data were measured from the slow-motion video frames of Casio Exilim FH20 camera.

**Figure 5:**
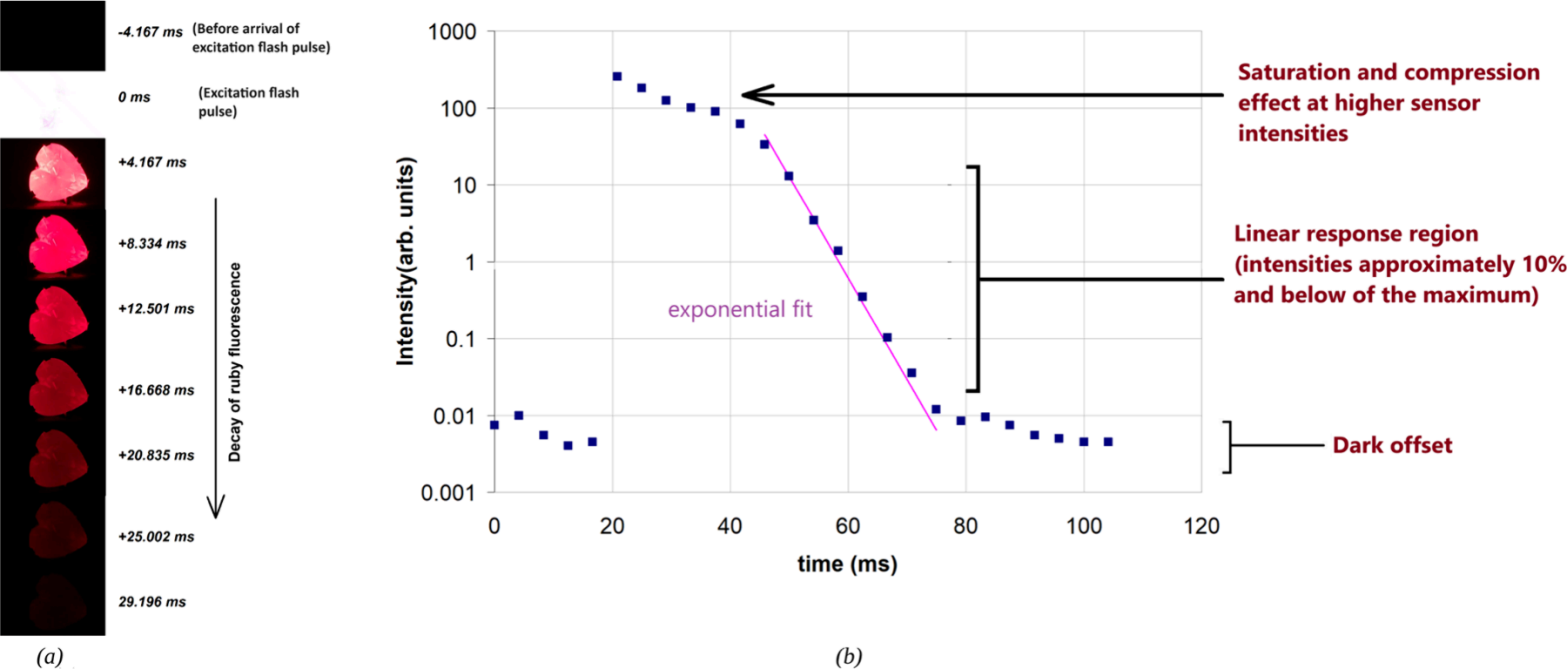
(a) Emission decay of ruby as captured in slow-motion video frames from a cellphone camera at 240 frames per second. A 40 mm focal length lens was used in front of the cellphone camera to get a better close up view of the ruby crystal. (b) Plot of emission decay profile after exciting the ruby sample with a photographic flash. The plotted emission intensity data were measured from the slow-motion video frames recorded using a cellphone camera. Please note that the vertical axis is on a logarithmic scale to better represent the data and the compression and saturation effects in cellphone camera video frames.

Figures 3(a), 4(a), and 5(a) show the individual frames arranged as a function of decay time for the ZWO ASI662MC, Exilim FH20, and cellphone camera (Google Pixel 4) slow-motion videos, respectively. The videos corresponding to the decay recorded from all three cameras can be seen in the Supporting Information section (Videos S1, S2, and S3).

### Measuring Emission Decay Time from the Video Frames

Figures 3(b), 4(b), and 5(b) show typical ruby emission decay profiles as measured from the intensity of ruby emission in the slow-motion video frames from ZWO ASI 662MC, Casio Exilim FH20, and cellphone (Google Pixel 4) cameras, respectively. In order to compute the intensities, the video frames were imported into ImageJ, and a region of interest (ROI) was created to enclose the ruby crystal. The average intensity inside the selected ROI for every frame was then measured, and a plot of average intensity as a function of time was made. The time between each frame is determined by the frame rate of the slow-motion video recording (for example, 1000 or 240 frames per second). For ZWO ASI662MC camera, the frame time can be directly extracted from the.fits frame metadata. Detailed step-by-step instructions for data analysis are available in the Supporting Information. An ImageJ macro, along with a comprehensive student handout containing step-by-step instructions for data processing, has also been provided in the Supporting Information and can be utilized for fast and easy data analysis (ImageJ Macro files, Comprehensive student handout, and Figures S1–S12 therein). Videos showing all of the data analysis steps are also available in the Supporting Information section (Experiment Video 1 and Experiment Video 2).

Figure 3(b) shows a typical ruby emission decay profile measured using an ASI662MC camera. The time constant calculated was approximately 4.5 ms, obtained by fitting a single exponential decay profile to the data. The fit was computed using the curve fitting function in ImageJ. Figure 4(b) shows the decay profile measured using a Casio Exilim FH20 camera with videos shot at 1000 frames per second. The profile shown in Figure 4(b) is an average of ten different measured decays. The average emission decay time measured was 3.55 ms with a standard deviation of 0.06 ms. Figure 5(b) shows the decay profile measured from a cellphone camera frame; it must be noted that the vertical axis is in the logarithmic scale to better represent the data. Here we see that the higher intensities deviate from the single exponential decay. This is attributed to the fact that the in the video frames recorded by the cellphone camera and Casio Exilim are compressed (e.g., JPEG compression of the frames) and do not contain raw data. This affects the accurate computation of the decay times, as compression may alter the intensity values of pixels in the frame. This compression effect is in addition to the inherent nonlinearities of the CMOS sensor.

A workaround to minimize compression artifacts in the data recorded by the cellphone and Casio Exilim camera, based on several experiments, was to keep the peak intensity below 10% of the maximum value. For example, if the maximum possible intensity before saturation is 255 (8 bits per video per color channel), then keeping the intensity in the region of interest well within 25 counts provided reasonably accurate data (for example, Figures 4(b) and 5(b)). The exponential fit made on the intensities below 10% of the maximum value for the data recorded using cellphone camera (Figure 5(b)) gives a decay time constant of 3.6 ± 0.3 ms for the ruby emission decay. Another way to avoid compression artifacts is simply by using a camera that can record raw video frames, such as amateur astronomy cameras like the one used here. Frame compression-induced artifacts in the intensity readings are not an issue for the ASI662MC camera, as it can record raw data frames.

A comparison of measured values from three different slow-motion digital cameras and a representative value reported in the literature is shown in Table 1. It is to be noted that ruby lifetimes can vary broadly depending on the specifics of the sample (chromium ion concentration, size of the crystal etc.). The measured values, particularly using the ZWO ASI662MC camera, are close to the reported room temperature decay time values reported in the literature.^2,3^ Because raw frames were recorded, the data from the ZWO ASI 662 MC camera are likely closer to the accurate value, as shown in Table 1. The slightly longer decay time measurement is attributed to the size of the ruby crystal used in the experiments (∼6.5 mm), due to reabsorption and re-emission of the emitted light (also known as radiation trapping).^18,24^ As the size of ruby crystal increases, some of the emitted light may be reabsorbed in the crystal and then re-emitted. This may result in an apparent increase in the lifetime when fitting a single exponential decay and can also cause a slight deviation of the measured decay profile from a pure single exponential decay.^18^

**Table 1:** Measured Time Constant of Ruby Emission Decay with Different Slow-Motion Cameras

Camera system	Frames per second	Time constant (ms)	Rate constant (s^–1^)	Reference	Raw frames
Cellphone Camera (Google Pixel 4)	240	3.6 ± 0.3	278 ± 25	This work	No
Casio Exilim FH20 HS	1000	3.55 ± 0.06	280.9 ± 4.2	This work	No
ZWO ASI 662MC	∼ 530	4.38 ± 0.15	228.5 ± 8.0	This work	Yes
Representative literature values		4.268 ± 0.006		ref (3) (Epsoti et al.)	

The emission lifetime calculated from Casio Exilim and cellphone camera data showed an increased deviation from the typical reported values in the literature (Table 1). This is primarily attributed to the compression of video frames, frame rate fluctuation and uncertainties, and to a lesser extent, the nonlinear response of the CMOS camera sensor.

From the recorded data, students can also determine the parameters of exponential decay, such as the rate constant and lifetime. The demonstration video (Experiment Video 2) in the Supporting Information details how to fit an exponential decay curve to the recorded data using ImageJ. Additionally, it is also possible to carry out measurements at different temperatures of the samples to monitor how temperature, concentration of Cr^3+^ ions, size of ruby crystal, etc. affect the emission decay rate constant. It is to be noted that users may want to try different fits on their own samples (such a biexponential fit) and may find residuals to see which fit is better.

Spectral analysis of emission decay as a function of time can be done by replacing the camera lens with a spectroscope and keeping rest of the experimental setup largely the same. (Figure S13 in the Supporting Information file discussing spectral analysis and IRF). Also, the instrument response can be measured by turning the flash on without the ruby sample present (Figure S14 in the Supporting Information file discussing spectral analysis and IRF).

## Conclusion

It has been shown here that a photographic flash and a slow-motion camera can be used to visualize the emission decay from a ruby crystal, and the ruby crystal’s metastable state lifetime can be calculated from analyzing the video frames. When raw frames are used for computation, the calculated decay lifetimes closely match other reported values in the literature. This demonstration provides a safe and easy way for students in advanced undergraduate courses or advanced high-school curricula to better understand emission decay, kinetics, and related concepts. As lasers become an integral part of many chemistry courses, this demonstration also has educational value for students to better grasp the fundamentals of lasers and their applications.

## Hazard Statement

There are no known significant hazards associated with this demonstration per the author’s knowledge. However, the following precautions must be taken during the conduct of the experiment:

1. Ensure that the photographic flash unit is in a good working condition and make sure that the outside enclosure of the flash unit is not broken or compromised. If so, use a different flash unit. There are high voltages inside a flash unit, and a broken outside enclosure could potentially expose one to the high voltages.
2. Even though low power flash use is generally known to be safe, do not stare directly at the flash emission to avoid any possibility of temporary visual discomfort or afterimages.

## Learning Objectives and Suggestions

### Learning Objectives

This experimental demonstration can be used toward several learning objectives. For example, this demonstration can help students learn the kinetic law of fluorescence decay. By visually observing the decay, mathematically fitting the exponential decay curve to the data, and computing the decay rate and lifetime, students will strengthen their understanding of these important chemistry concepts.

In addition, this demonstration can be useful while teaching the concepts related to long-lived metastable states, population inversion, and lasers. For example, measuring the milliseconds-long lifetime can help clarify to students the long-lived nature of the metastable state, which can allow for the buildup of the population in that state under strong optical pumping. Under sufficiently strong optical pumping, this buildup of population ultimately can result in achieving the population inversion condition(in ruby, gain medium of world’s first laser, this is between metastable state and the ground state) and lasing action in a three level system.

This demonstration can also be useful when teaching concepts related to fluorescence lifetime imaging. For example, printer paper can be pasted behind the ruby sample during the demonstration, and the emission from the blank paper can be measured followed by the measurements from the ruby sample. The emission from the paper will decay very fast (in 1–2 video frames), whereas emission from ruby will decay slowly. CRT phosphors with different decay characteristics can also be used for this effect instead of paper if the paper decay is too fast. This can show students how lifetime measurements can act as a contrast mechanism in microscopy and imaging.

### Suggestions for Classroom Use

For classroom use of this demonstration, the simplest approach could be for the instructor to show the demonstration, followed by students repeating the experiment and performing the data analysis.

A more involved approach would be to allow students to construct the setup (e.g., with 3D printed parts) after the initial demonstration by the instructor and let them carry out the experiment either individually or in teams with the provided samples. To make it more interesting, different ruby samples (for example, varying in size or concentration of Cr^3+^ ions) could be provided to different teams. A study could then be prepared from the measurements made by different teams, depicting the effect of crystal size, Cr^3+^ ion concentration, or any other parameter, such as temperature, on ruby emission decay kinetics. A short handout with questions that could be asked of students about what they observed in the demonstration is included in the Supporting Information file with the handout of questions.

## Supporting Information Available

Video S1: Ruby emission decay frames from Casio Exilim camera (AVI)
Video S2: Ruby emission decay frames from Google Pixel 4 cellphone camera (AVI)
Video S3: Ruby emission decay frames from ZWO ASI662MC camera (AVI)
STL files for 3D printing the experimental setup (ZIP)
ImageJ Macro files (ZIP)
Experiment video 1: Demonstration video showing experimental setup, data recording and analysis (MP4)
Experiment video 2: Demonstration showing data analysis using the ImageJ macro (MP4)
Comprehensive student handout: Step by step instructions for experiment and data analysis, including the code (PDF)
Spectral analysis and IRF measurements on the ruby sample (PDF)
Short handout of questions that can be asked from students (PDF)

## Supplementary Material

ed4c01529_si_001.avi

ed4c01529_si_002.avi

ed4c01529_si_003.avi
