# Supplementary material for: Visualizing Ruby Emission Decay Lifetime with Slow-Motion Digital Cameras: A Demonstration for Students
Source: J Chem Educ. 2025 Apr 7;102(5):2252–8. doi: 10.1021/acs.jchemed.4c01529 (PMC12080249; doi:10.1021/acs.jchemed.4c01529)
Supplement: Supplementary file 8 — ed4c01529_si_008.pdf [file ed4c01529_si_008.pdf]

Supporting Information

Visualizing ruby emission decay lifetime with slow motion digital cameras : A demonstration for students

Dinesh Dhankhar, Los Alamos National Laboratory, Los Alamos, New Mexico, USA 87545
dineshiist21@gmail.com

Comprehensive student handout

Outline

1. Some useful links and resources
    1.1. Experiment demonstration and data analysis videos
    1.2. Links for downloading the software

2. Step by step procedure to carry out the experiment
    2.1. Constructing the experimental setup
    2.2. Carrying out the experiment and recording the data

3. Step by step procedure for data processing
    3.1. Renaming video frames captured using ZWO ASI 662MC camera
    3.2. Installing macro on ImageJ
    3.3. Select the data
    3.4. Select region of interest (ROI) for further analysis
    3.5. Doing curve fitting
    3.6. Save the video to visualize the decay of fluorescence
    3.7. Finishing up
    3.8. Creating properly named fits files from Casio Exilim or cellphone camera videos

4. Appendix
    4.1. Macro code for processing data
    4.2 Guidance for buying ruby samples and other components.

1. Some useful links and resources

1.1. Experiment demonstration and data analysis videos

It would be useful to watch the following two videos to get a general overview of the experiment and data analysis steps :

Video 1 : Demonstration of experimental procedure and step by step data analysis
https://youtu.be/T4PBjxtAId4

Video 2 : Demonstration of data analysis steps using ImageJ macro
https://youtu.be/oC2L1UcoKos

The above two videos can also be downloaded from the supplementary information section of the paper.

1.2. Links for downloading the software

After getting an overview of the experiment and preparing to carry out your own experiment, it would be useful to download the software required for this demonstration. The software can be downloaded from the following links. All the software listed below is free to download and use.

1. ImageJ
   https://imagej.net/ij/download.html

2. PIPP
   https://web.archive.org/web/20230531163522/https://sites.google.com/site/astropipp/downloads

   https://www.rsastro.com/downloads/

3. SharpCap
   https://www.sharpcap.co.uk/sharpcap/downloads

4. Advanced Renamer
   https://www.advancedrenamer.com/download

2. Step by step procedure to carry out the experiments

2.1. Constructing the experimental setup

The experimental setup can be constructed using 3D-printed parts. The 3D model files (.stl) are provided in the supplementary information section of the paper. These .stl files can be opened in software like Creality Slicer to create 3D printer-readable G-code files for printing. After printing the files, the setup can be constructed using the brief instructions provided in Video 1 (https://youtu.be/T4PBjxtAId4). Printing the files with a 3D printer might take several hours; therefore, plan to print the files in advance.

2.2. Carrying out the experiment and recording the data

If using a ZWO ASI662MC camera, connect the camera to the computer using a USB 3.0 cable. Open Sharpcap software, find your camera listed in the camera dropdown menu, and select it to open the camera. If you do not find your camera listed, make sure you download and install the drivers for the camera. The drivers can be found using an online search or by using this link. (Software - Discovery Astrophotography with ZWO ASTRO).

Once the camera is ready, make the following settings in the Sharpcap software, as shown in the image below (Figure S1). Select the color space as RAW16 and the output format as .fits. Choose a custom capture area with a height of 100 pixels and a width of 200 pixels. Using the Pan and Tilt adjustments in the Region of Interest Selection, bring the image of your ruby sample within your 200x100 pixel capture area.

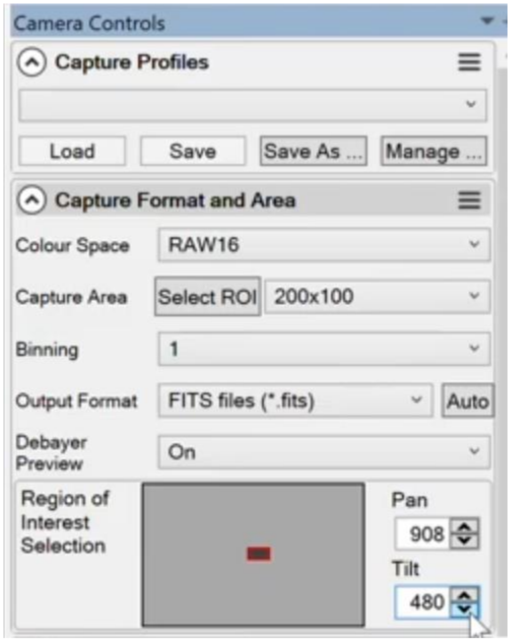

Figure S1. Camera Settings on the Sharpcap software

Bring your ruby sample image to a sharp focus in the image by rotating the lens focusing ring on the front of the camera. You may increase the camera gain to get a clear view of the ruby sample.

After having the ruby image focused, and centered in your custom capture area, reduce the gain to a low value, such as 1. Set exposure time to a low value, such as 0.2 milliseconds. After doing that, you may be

able to see the frame rate at the bottom information ribbon on the software. Make sure that the frame rate is ~ 500 fps (see Figure S2).

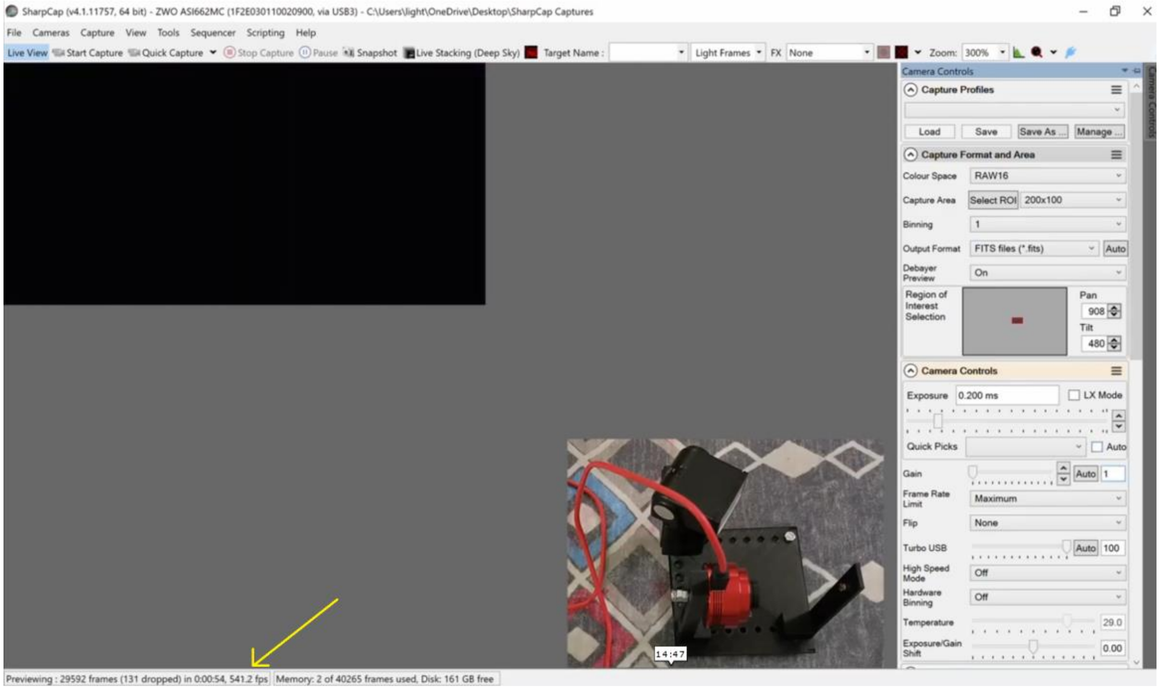

Figure S2. Camera settings and ensuring the frame rate is sufficiently high for the experiments

Turn on your photographic flash light and make sure that flash is ready for triggering (e.g. either by pressing a button on the flash unit or remotely). Select down arrow on the right of quick capture menu, click on unlimited, trigger your photographic flash and then click on stop capture button. The captured frames will then be recorded into the computer memory.

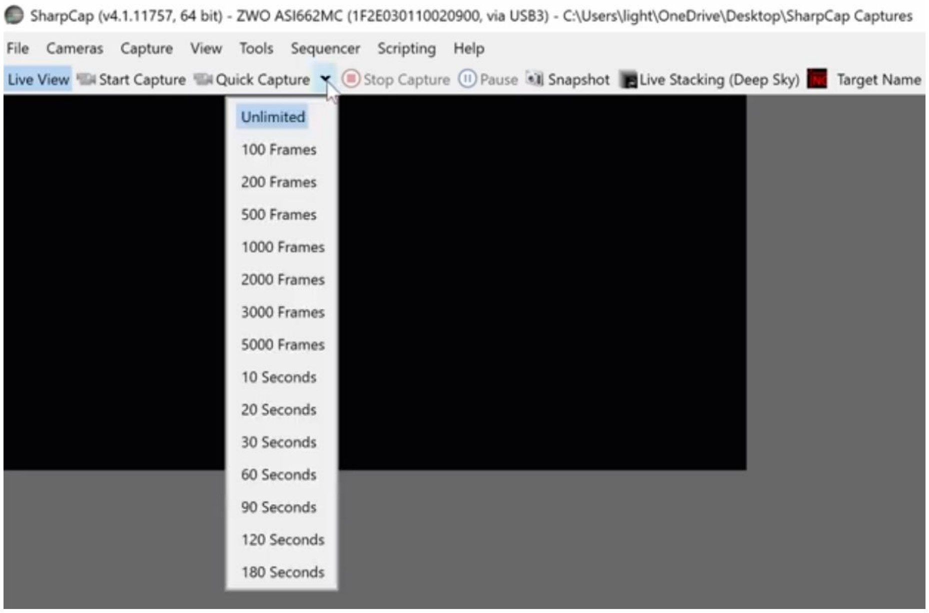

Figure S3. Starting capture of frames in the sharpcap

If using a cellphone camera to capture the slow motion video, select 1/8x slow motion setting, which is ~ 240 frames per second capture rate. You may also use a closeup lens in front of the cellphone camera to get a better close up view of your ruby sample. Start video capture, trigger your flash, and stop the video capture. Transfer the captured video file to the computer for further data processing.

If using Casio Exilim FH20 camera, capture the video in 1000fps mode. Focus on the ruby sample, and reduce the ISO setting to the lowest possible number (100).

3. Step by step procedure for data processing

3.1. Renaming video frames captured using ZWO ASI 662MC camera

Go to the folder containing the captured image frames. The text file, in the folder may be deleted or saved somewhere else. Load all the .fits image frames in the Advanced renamer software. On the advanced renamer, use the following settings to rename the frames. Click, Start batch, and that would rename the files to the

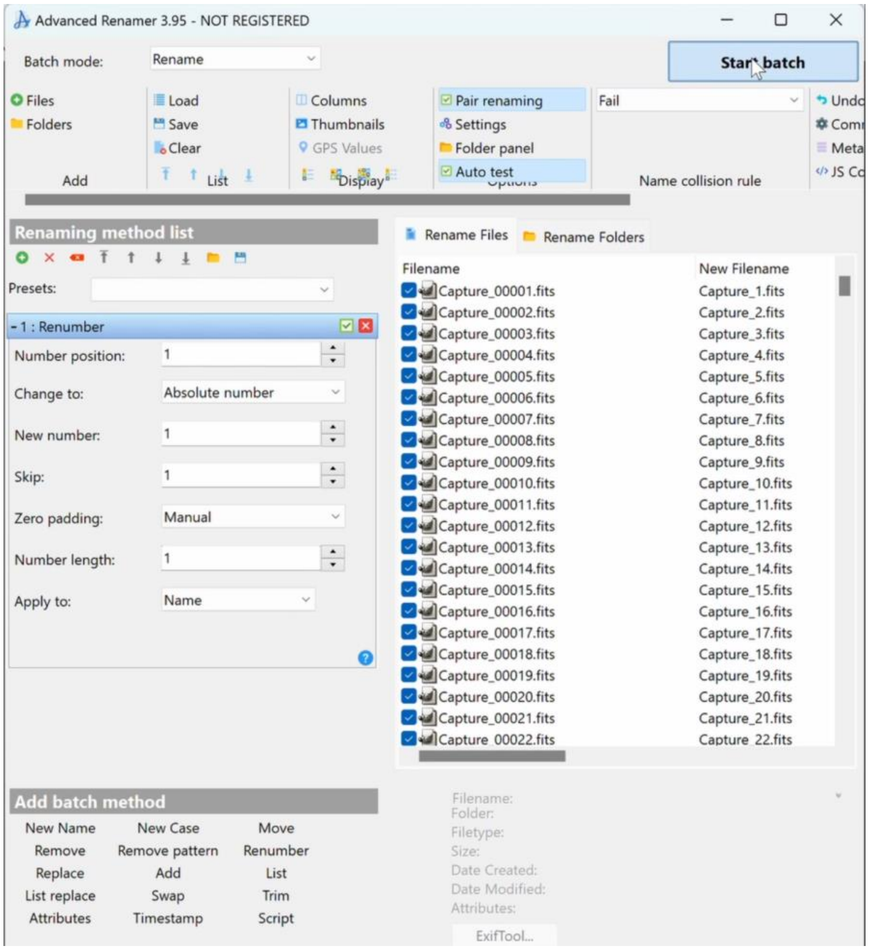

Figure S4. Advanced Renamer settings

3.2. Installing macro on ImageJ

On ImageJ, go to Plugin→ Install→ and select the file “Macro_ruby_v3.ijm”. This file is provided in the supplementary information section. Code in this macro file is also provided in the appendix section of this document, so you can modify the file if required. You may also watch the Video 2 regarding installing the macro on ImageJ. After the macro is installed, you can see a prompt with file name when you place cursor on the macro menu (see Figure S5).

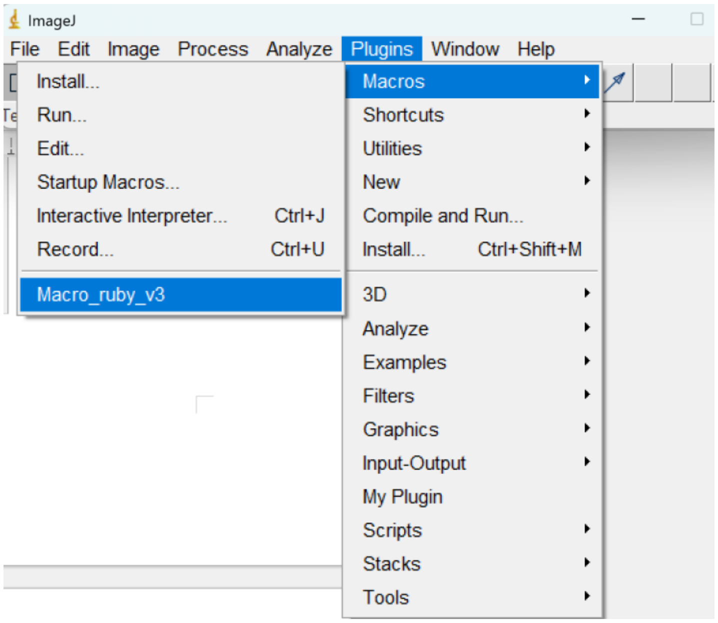

Figure S5. Installed macro

3.3. Select the data

Once you click on the installed macro, it will take you to a pop up screen and prompt to select the folder where your renamed files are stored. You will also need to enter the number of image frames are there in the folder (See Figure S6).

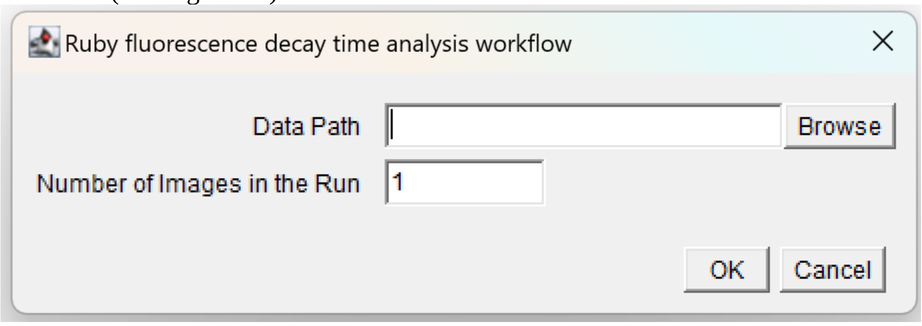

Figure S6. Initial prompt in the macro

Once you click ok, it will analyze all the images in the folder to find the frames where the flash pulse arrived.

3.4. Select region of interest (ROI) for further analysis

After all the images are analyzed, the macro will identify the frames where the flash pulse arrived and show you one of the subsequent images asking to select a region of interest enclosing the ruby crystal Figure S7.

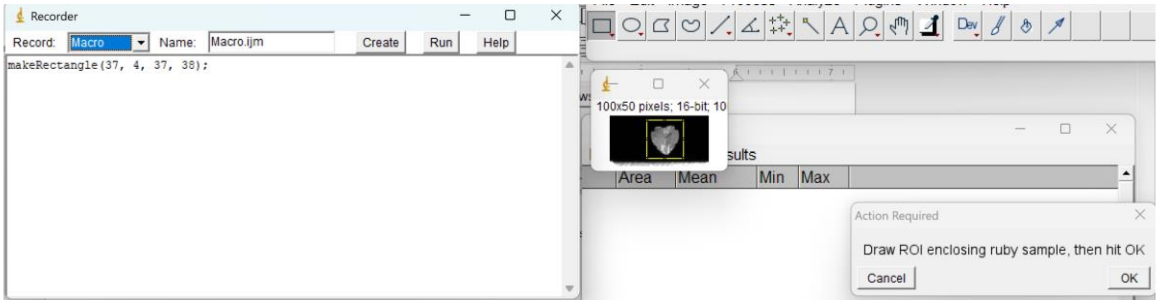

Figure S7. Selecting region of interest for image intensity analysis

3.5. Doing Curve fitting

Macro would extract the intensities in the selected region of interest along with their frame times, and print them on a log file that would be printed on the screen. The same data would also be written in a Results folder in the folder containing the original images in the text and excel files. A curve fitter prompt would be opened. Select and copy-paste the data from the log file in to the curve fitter. You may select the data starting from the subsequent to the maximum intensity to obtain a proper exponential fit. See Figure S8 below for the details.After the fit is made, click OK on the prompt and a screenshot of the fit plot would be saved in the Results folder.

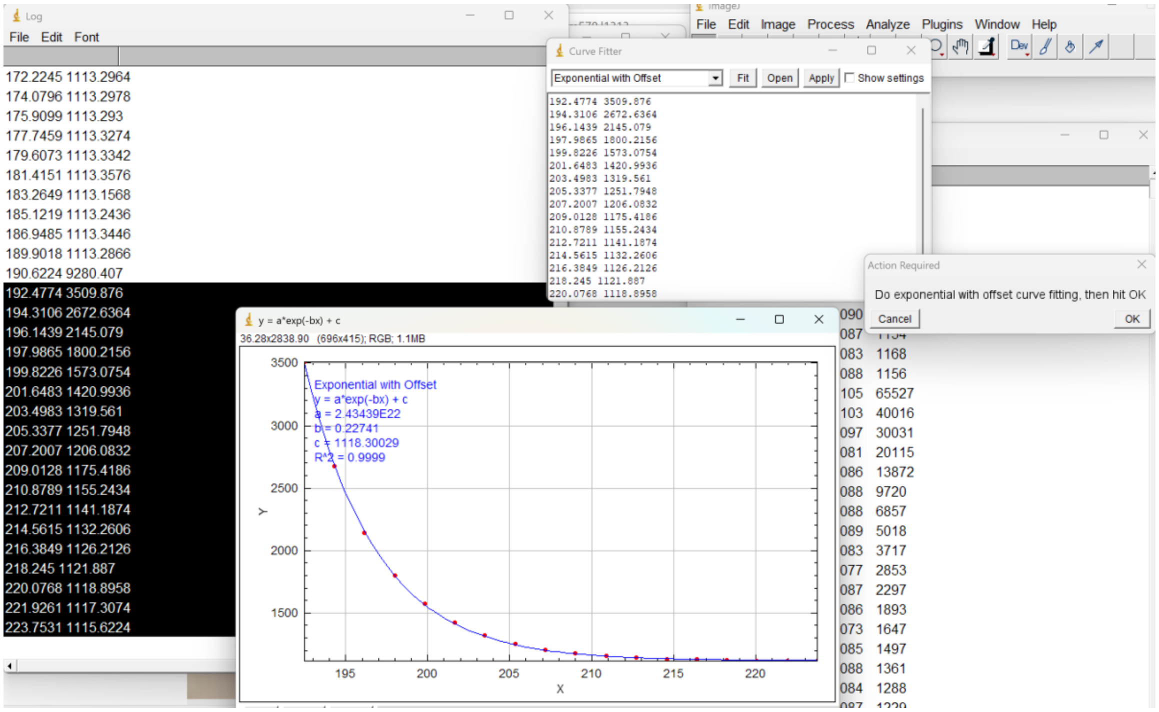

Figure S8. Making a curve fit

3.6. Save the video to visualize the decay of fluorescence

The next prompt will ask you to save an avi video file built with the image frames around the flash pulse excitation. Yo can chose the type of compression and the frame rate for this video to play.

3.7. Finishing up

After you are done saving the video, close any other open prompts in ImageJ.

3.8 Creating properly named fits files from Casio Exilim or cellphone camera videos

Please note that because of data compression used in cellphone and Casio Exilim videos, the results will be far less accurate as compared to the results obtained with ZWO ASI662MC camera, where raw video frames can be recorded.
Copy the cellphone or Casio Exilim recorded video to the computer. Drag and drop this video file to the PIPP 'Source Files' tab (Figure S9). In the 'Input Options' tab on PIPP, select 'Input Frame Colour/Monochrome' as Colour (Figure S10).
In the 'Processing Options' tab on PIPP, select the checkbox next to 'Convert Colour To Monochrome' and select 'Use R Channel Only' from the dropdown menu (Figure S11). In the 'Output Options' tab, select Output format as FITS, Output Filename Prefix as 'Capture_' and leave Output Filename Suffix as blank. Also uncheck all other options from FITS File Naming (Figure S12).

After making the above mentioned settings, on PIPP. select 'Do Processing' tab on PIPP, and click 'Start processing'. This will make a folder with properly named files in a new folder.

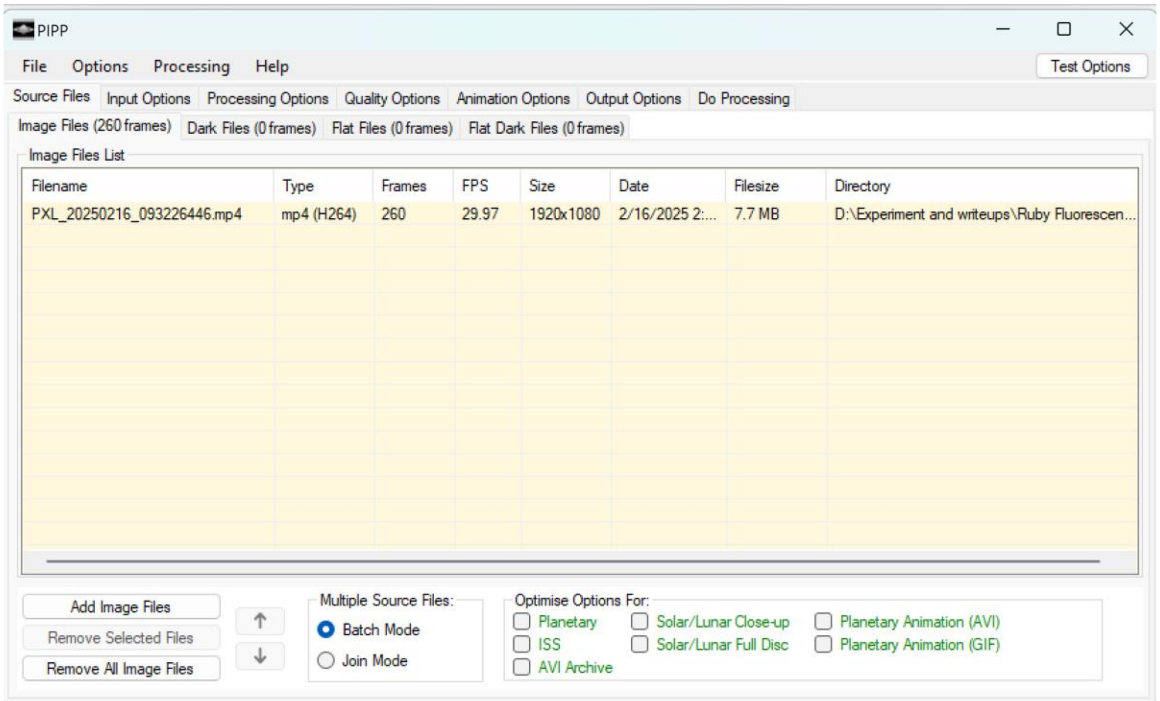

Figure S9. Source files tab on PIPP.

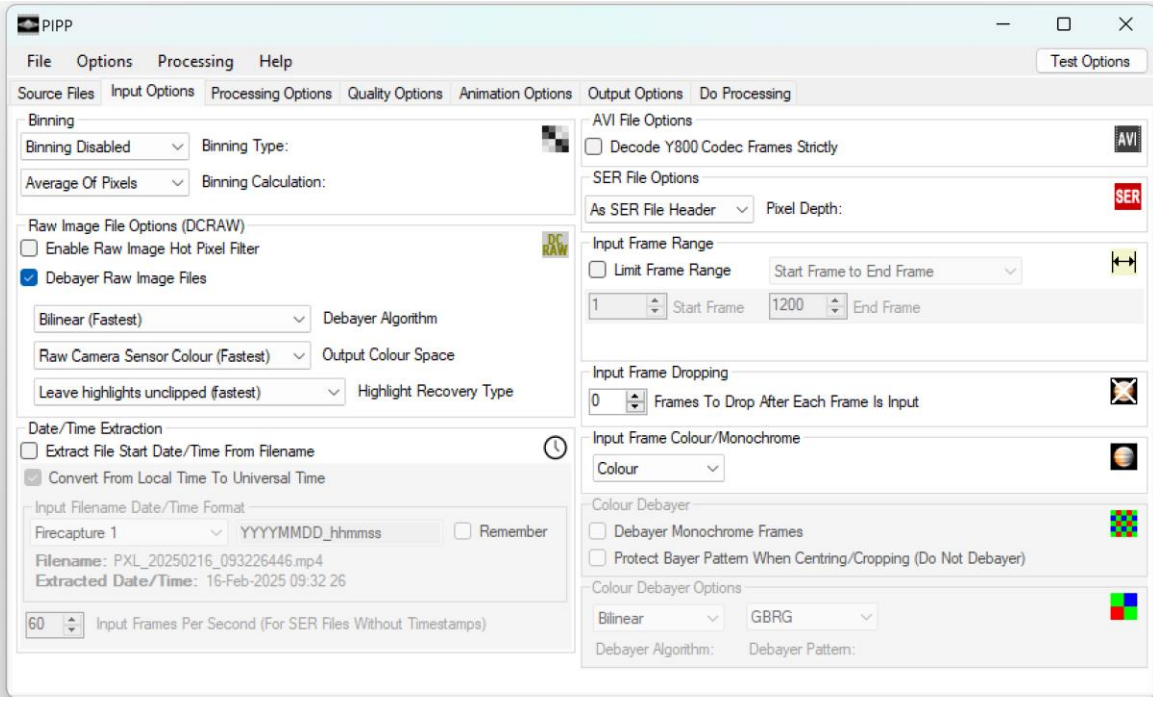

Figure S10. Input options settings on PIPP

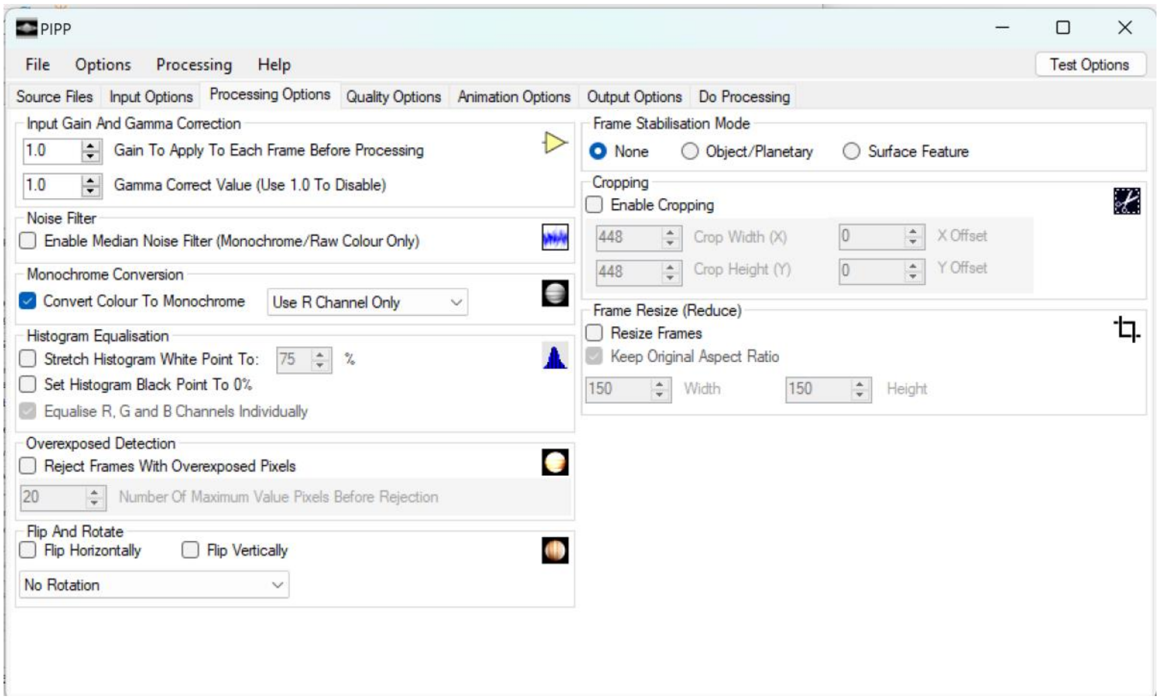

Figure S11. Processing options settings on PIPP

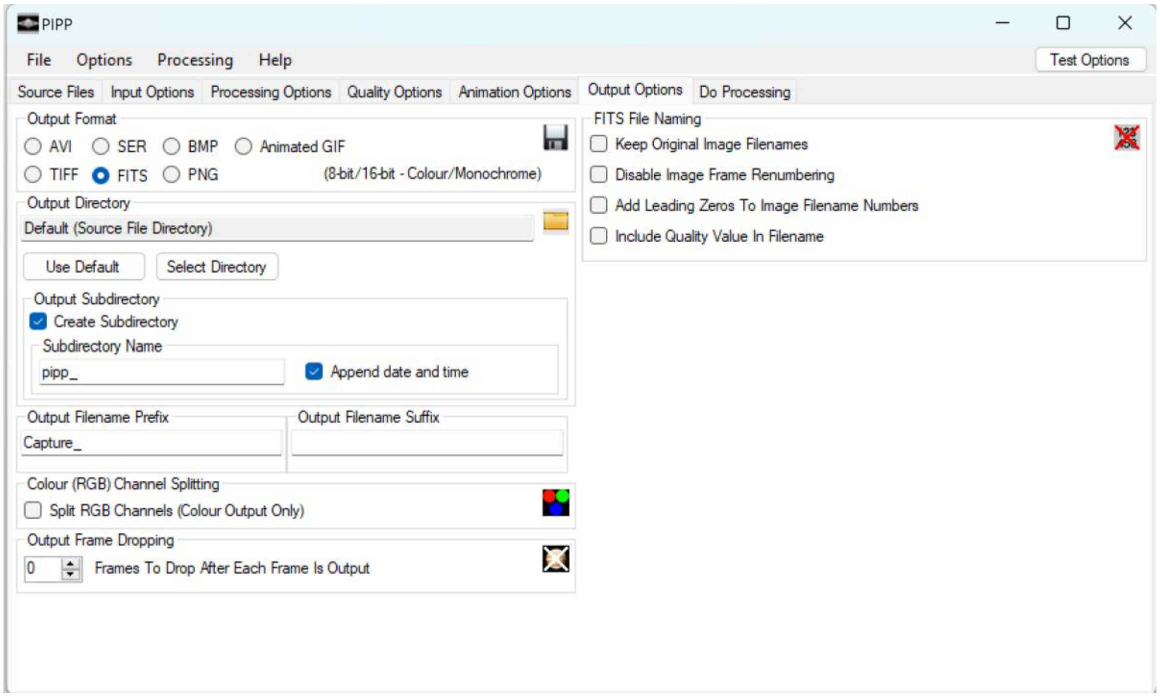

Figure S12. Output options settings on PIPP

In the next step, install macro on ImageJ, macro_ruby_v4_casio.ijm for Casio Exilim FH20 videos, and macro_ruby_v5_cellphone.ijm for cellphone camera slow motion videos. Rest of the steps are the same as from steps 3.3 to 3.7, as described before in this section.

4. Appendix

4.1. Macro code for processing data

From ZWO ASI 662MC Camera

```
run("Clear Results");
data_path=getDirectory("image");
Dialog.create("Ruby fluorescence decay time analysis workflow");
Dialog.addDirectory("Data Path", data_path)
Dialog.addString("Number of Images in the Run", "1");
Dialog.show();

data_path = Dialog.getString();
n = Dialog.getString();

//create results directory

results_dir =data_path+"\\Results\\";
if(!File.exists(results_dir))
{
File.makeDirectory(results_dir);
}

i =1;
b = 1;
max_intensity_frame = 1;
c=1;
d =1;
t_ms =1;
```

```
// 1. Measure all frames average intensity
for(i=1;i<n;i++){
open(data_path+"/Capture_"+i+".fits");
selectImage("Capture_"+i+".fits");
run("Measure");
close;
}

//2. Find the frames where pulse arrived
for(i=1;i<n-1;i++){
b = getResult("Mean", i);
if (b > getResult("Mean", max_intensity_frame))
{
max_intensity_frame = i;
}
}
run("Clear Results");

//3. select ROI on these frames
count = 1;
e=1;
open(data_path+"/Capture_"+(max_intensity_frame+2)+".fits");
run("Bin...", "x=2 y=2 bin=Average");
run("Record...");
waitForUser("Draw ROI enclosing ruby sample, then hit OK");
roiManager("Add");
roiManager("Select", 0);
close;

//4. print time vs intensity and do the curve fitting
print("time(ms)","Intensity");
for(i=max_intensity_frame-10; i<max_intensity_frame+20;i++){
open(data_path+"/Capture_"+i+".fits");
wait(10);
b=getInfo("DATE-AVG");
c = split(b, "..");
d = split(c[1], "'.");
selectImage("Capture_"+i+".fits");
run("Bin...", "x=2 y=2 bin=Average");
roiManager("Select", 0);
wait(10);
run("Measure");
e = getResult("Mean", count-1);
print(parseFloat(d[0])/10000,e);
count = count +1;
close;
}
selectWindow("Log");
saveAs("Text", results_dir+"/Decay_data.txt");
saveAs("Text", results_dir+"/Decay_data.xls");

run("Curve Fitting...");
waitForUser("Do exponential with offset curve fitting, then hit OK");
selectImage("y = a*exp(-bx) + c");
saveAs("Jpeg", results_dir+"/fit_plot.jpg");

while (nImages>0) {
        selectImage(nImages);
        close();
    }

//5. Save avi video for visualization
for(i=max_intensity_frame-10; i<max_intensity_frame+20;i++){
open(data_path+"/Capture_"+i+".fits");
run("Bin...", "x=2 y=2 z=1 bin=Average");
```

```
}

run("Images to Stack", "name=video use keep");
run("AVI... ");

//6. close all open images
while (nImages>0) {
        selectImage(nImages);
        close();
    }
```

From Casio Exilim FH20 camera (at 1000 fps)

```
run("Clear Results");
data_path=getDirectory("image");
Dialog.create("Ruby fluorescence decay time analysis workflow");
Dialog.addDirectory("Data Path", data_path)
Dialog.addString("Number of Images in the Run", "1");
Dialog.show();

data_path = Dialog.getString();
n = Dialog.getString();

//create results directory

results_dir =data_path+"\\Results\\";
if(!File.exists(results_dir))
{
File.makeDirectory(results_dir);
}


i =1;
b = 1;
max_intensity_frame = 1;
c=1;
d =1;
t_ms =1;


// 1. Measure all frames average intensity
for(i=1;i<n;i++){
open(data_path+"/Capture_"+i+".fit");
selectImage("Capture_"+i+".fit");
run("Measure");
close;
}

//2. Find the frames where pulse arrived
for(i=1;i<n-1;i++){
b = getResult("Mean", i);
if (b > getResult("Mean", max_intensity_frame))
{
max_intensity_frame = i;
}
}
run("Clear Results");

//3. select ROI on these frames
count = 1;
e=1;
open(data_path+"/Capture_"+(max_intensity_frame+2)+".fit");
run("Bin...", "x=2 y=2 bin=Average");
run("Record...");
waitForUser("Draw ROI enclosing ruby sample, then hit OK");
```

```
roiManager("Add");
roiManager("Select", 0);
close;

//4. print time vs intensity and do the curve fitting
print("time(ms)","Intensity");
for(i=max_intensity_frame-10; i<max_intensity_frame+40;i++){
open(data_path+"/Capture_"+i+".fit");
//b=getInfo("DATE-AVG");
//c = split(b, "..");
//d = split(c[1], "'.");
selectImage("Capture_"+i+".fit");
run("Bin...", "x=2 y=2 bin=Average");
//makeRectangle(8, 2, 66, 37); //paste ROI here
roiManager("Select", 0);
run("Measure");
e = getResult("Mean", count-1);
print(count,e);
count = count +1;
close;
}
selectWindow("Log");
saveAs("Text", results_dir+"/Decay_data.txt");
saveAs("Text", results_dir+"/Decay_data.xls");

run("Curve Fitting...");
waitForUser("Do exponential with offset curve fitting, then hit OK");
selectImage("y = a*exp(-bx) + c");
saveAs("Jpeg", results_dir+"/fit_plot.jpg");

while (nImages>0) {
        selectImage(nImages);
        close();
    }

//5. Save avi video for visualization
for(i=max_intensity_frame-10; i<max_intensity_frame+20;i++){
open(data_path+"/Capture_"+i+".fit");
run("Bin...", "x=2 y=2 z=1 bin=Average");
}

run("Images to Stack", "name=video use keep");
run("AVI... ");

//6. close all open images
while (nImages>0) {
        selectImage(nImages);
        close();
    }
```

From Cellphone camera (assuming 240 fps)

```
run("Clear Results");
data_path=getDirectory("image");
Dialog.create("Ruby fluorescence decay time analysis workflow");
Dialog.addDirectory("Data Path", data_path)
Dialog.addString("Number of Images in the Run", "1");
Dialog.show();

data_path = Dialog.getString();
n = Dialog.getString();

//create results directory

results_dir =data_path+"\\Results\\";
if(!File.exists(results_dir))
{
```

```
File.makeDirectory(results_dir);
}


i =1;
b = 1;
max_intensity_frame = 1;
c=1;
d =1.0;
t_ms =1;


// 1. Measure all frames average intensity
for(i=1;i<n;i++){
open(data_path+"/Capture_"+i+".fit");
selectImage("Capture_"+i+".fit");
run("Measure");
close;
}

//2. Find the frames where pulse arrived
for(i=1;i<n-1;i++){
b = getResult("Mean", i);
if (b > getResult("Mean", max_intensity_frame))
{
max_intensity_frame = i;
}
}
run("Clear Results");

//3. select ROI on these frames
count = 1;
e=1;
open(data_path+"/Capture_"+(max_intensity_frame+2)+".fit");
run("Bin...", "x=2 y=2 bin=Average");
run("Record...");
waitForUser("Draw ROI enclosing ruby sample, then hit OK");
roiManager("Add");
roiManager("Select", 0);
close;

//4. print time vs intensity and do the curve fitting
print("time(ms)","Intensity");
for(i=max_intensity_frame-10; i<max_intensity_frame+20;i++){
open(data_path+"/Capture_"+i+".fit");
//b=getInfo("DATE-AVG");
//c = split(b, "..");
d = count*(1000/240);
selectImage("Capture_"+i+".fit");
run("Bin...", "x=2 y=2 bin=Average");
//makeRectangle(8, 2, 66, 37); //paste ROI here
roiManager("Select", 0);
run("Measure");
e = getResult("Mean", count-1);
print(d,e);
count = count +1;
close;
}
selectWindow("Log");
saveAs("Text", results_dir+"/Decay_data.txt");
saveAs("Text", results_dir+"/Decay_data.xls");

run("Curve Fitting...");
waitForUser("Do exponential with offset curve fitting, then hit OK");
selectImage("y = a*exp(-bx) + c");
saveAs("Jpeg", results_dir+"/fit_plot.jpg");
```

```
while (nImages>0) {
        selectImage(nImages);
        close();
    }

//5. Save avi video for visualization
for(i=max_intensity_frame-10; i<max_intensity_frame+20;i++){
open(data_path+"/Capture_"+i+".fit");
run("Bin...", "x=2 y=2 z=1 bin=Average");
}

run("Images to Stack", "name=video use keep");
run("AVI... ");

//6. close all open images
while (nImages>0) {
        selectImage(nImages);
        close();
    }
```

4.2. Guidance for buying ruby samples and other components

Synthetic ruby samples can be bought rather inexpensively. Synthetic ruby jewelry is available on websites such as etsy or ebay, (typically under $50) whereas synthetic ruby spheres of different sizes can be purchased from suppliers such as Edmund Optics, or on ebay.

https://www.ebay.com/itm/115120852237

https://www.edmundoptics.com/f/sapphire-and-ruby-ball-lenses/12451/?srsltid=AfmBOorVPmJj01OLgkP_WhEF4qiDsMM7q1j2-J1MFA-hyTTqpBceaHv8

Astronomy camera used in this demonstration (ZWO ASI 662MC) is also inexpensive. As of this writing it cost around $ 149 on High Point Scientific website. Though the price frequently vary for this camera.

https://www.highpointscientific.com/zwo-asi662mc-usb-3-color-cmos-astronomy-camera-asi662mc
