# Supplementary material for: Visualizing Ruby Emission Decay Lifetime with Slow-Motion Digital Cameras: A Demonstration for Students
Source: J Chem Educ. 2025 Apr 7;102(5):2252–8. doi: 10.1021/acs.jchemed.4c01529 (PMC12080249; doi:10.1021/acs.jchemed.4c01529)
Supplement: Supplementary file 9 — ed4c01529_si_009.pdf [file ed4c01529_si_009.pdf]

Supporting Information

Visualizing ruby emission decay lifetime with slow motion digital cameras : A demonstration for students

Dinesh Dhankhar, Los Alamos National Laboratory, Los Alamos, New Mexico, USA 87545
dineshiist21@gmail.com

It is possible to measure the spectral decay of ruby fluorescence decay by attaching ASI662MC camera to the back of a spectroscope and using the same ruby sample and photographic flash. One such collected data is shown in the Figure S13. R1 and R2 emission lines of ruby fluorescence can be clearly seen, as well as broad fluorescence originating from direct transition from $^4T_2$ to $^4A_2$ transition (See Figure 1 in the main text for ruby energy levels). Interestingly, the broad fluorescence band appears to have the same fluorescence lifetime as ^2E to $^4A_2$ transition. This is reported to be due to two upper states being in thermal equilibrium with each other (Garland, C.W.; Nibler, J.W.; Shoemaker, D.P. Experiments in Physical Chemistry, 8th ed.; McGraw-Hill: New York, 2009; pp 484-492).

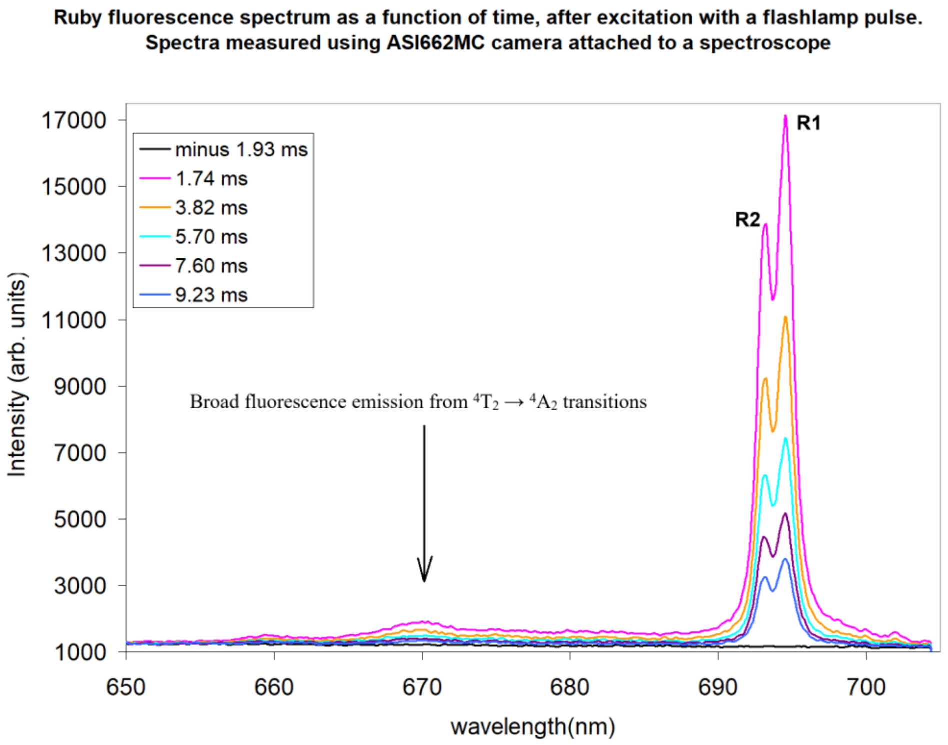

Figure S13. Ruby fluorescence spectrum as a function of time, after excitation with a short pulse photographic flash.

Figure S14 compares the instrument response when the flash is turned on with and without the ruby sample in the experimental setup. The plot shows that the flash turns off much faster than the decay time of the ruby.

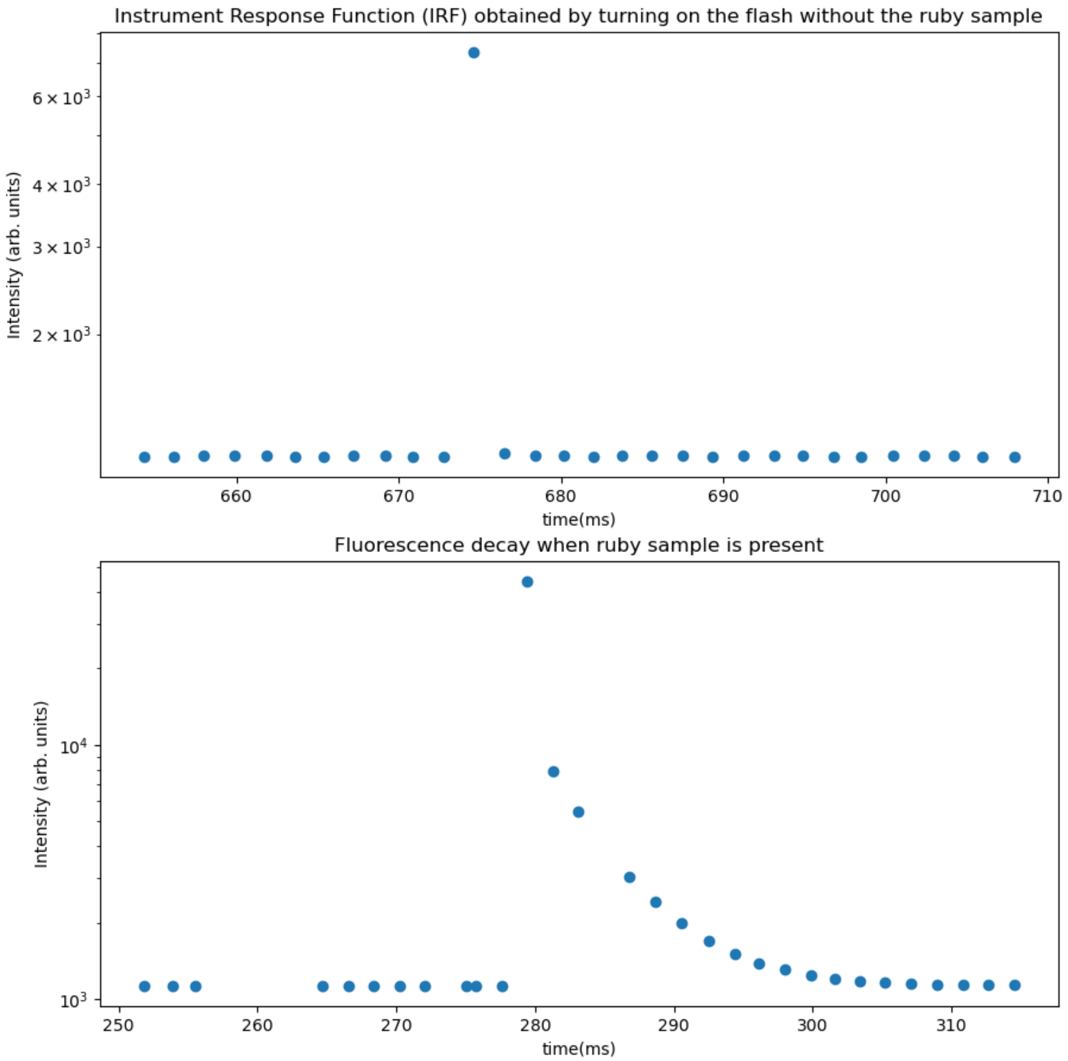

Figure S14. Top panel shows the measurements made when flash is turned on without the ruby sample present in the setup. This plot shows the instrument response function which can be utilized for deconvolution of the data. Bottom panel shows the decay plot when the ruby sample is present. The comparison show that the flash turns off much faster than the decay time of the ruby.
