# Supplementary material for: Visualizing Ruby Emission Decay Lifetime with Slow-Motion Digital Cameras: A Demonstration for Students
Source: J Chem Educ. 2025 Apr 7;102(5):2252–8. doi: 10.1021/acs.jchemed.4c01529 (PMC12080249; doi:10.1021/acs.jchemed.4c01529)
Supplement: Supplementary file 10 — ed4c01529_si_010.pdf [file ed4c01529_si_010.pdf]

Supporting Information

Visualizing ruby emission decay lifetime with slow motion digital cameras : A demonstration for students

Dinesh Dhankhar, Los Alamos National Laboratory, Los Alamos, New Mexico, USA 87545
dineshiist21@gmail.com

Handout of questions to ask students after the demonstration

1. What color does ruby fluoresce after excitation from the photographic flash ?
2. Did the ruby fluorescence decayed immediately ?
3. Could a mathematical function describe the intensity of fluorescence decay as a function of time ?
4. Can you explain the concept of decay constant and lifetime of fluorescence ?
5. Can you explain why the intensity of fluorescence decayed ?
6. Can you give some examples of industrial uses of ruby ?

For advanced students

7. Can you differentiate between spontaneous and stimulated emission ?
8. What would be the lifetime of stimulated emission ?
9. Can you describe working of a ruby laser ?
